# In Whose Eye Is the Mote?

**Published:** 1885-04

**Authors:** P. P. Wells


### IN WHOSE EYE IS THE MOTE?

Dear Homœopathic Physician:—In your issue for March, Vol. V, p. 107, I am charged by M. D. Lummis, M. D., with "rashness" for saying it gave me pleasure to hear of one college where the *Organon* was taught to its classes. I certainly was glad to hear this, though that college was so far away that I had had no opportunity to learn the fact from either teacher or pupils till I had spoken of *all* as equally deficient in this duty. Why it should be deemed "rash" by Dr. Lummis to express this gladness is wholly beyond my comprehension. Worcester defines "rash—*hasty, violent, precipitate.*" I fail to see either of these in my quiet and honest remark of the gladness the fact brought to me. In the same connection I inquired if there were another. I did not intend to do this "rashly," and I don't think I did. I intended no offense to any college or any friend of any college not guilty of this neglect. I further said, "If there be another, its whereabouts is unknown." This was certainly true when said, and I was not aware at the time of "haste," "violence," or "precipitation" in the utterance. But, says Dr. Lummis, the Boston University teaches the *Organon*, and "teaches it well." * If I may be allowed, without charge of "rashness,"

* Perhaps we should beg pardon of Dr. Lummis for entertaining even a slight suspicion as to the character of the result of this teaching in the acquired knowledge of the student. If we have such suspicion he alone is responsible for it. He says: "The undergraduates are 'quizzed' upon its teaching, thus giving them a double chance to learn its strength and weakness," etc. Then these students have special pains spent on them to teach them the "weaknesses" of our organic law, and doubtless they think, "*Now we know them.*" Our difficulty with this is not that these teachers or their pupils may attribute "weaknesses" to our codified law—this they may do if it be the best they can attain to in their knowledge of it—but that neither the "*strength*" nor the "*weakness*" of the philosophy of the *Organon* can be so, and there taught and learned. These can only be demonstrated in the clinical experience of intelligent, earnest, and honest prescribers. We cannot forget that not seldom has prejudiced strabismus been able to discern only weaknesses in its most important truths. Indeed, so damaging is this misfortune that its afflicted subjects have been often found wholly incapable of distinguishing "strength" from "weakness." *How much* of this university teaching or "quizzing" has been spent on supposed "weaknesses," and what were the reasons for so judging them? May they not possibly be strength, rather?

Our first introduction to one of our ambitious neighbors was to listen to a paper he had written on the "Errors and Fallacies of Hahnemann." He discoursed fluently and confidently on these, just as though there were many, and he knew all about them. When questioned, at the close of his reading, he was compelled to admit he knew not one. We have an habitual suspicion of all who talk of these "errors" and "fallacies" and "weaknesses," *assuming* these to be facts when they know nothing about them.

I will say I am glad of that, too. And with the same reserve, I will say I was glad to hear there was another college west of us where this code of God's therapeutics was *sometimes* mentioned; and more, that it had been honored with *one* lecture devoted to its consideration some time during the session. This is certainly better than nothing, and may not one be a little glad of it and escape the charge of rashness? But there is more and better. Another college, by the mouth of one of its faculty, has declared, since this charge of neglect, "though true heretofore, it shall not be hereafter." And still another, at the beginning of its session, announced that the *Organon* would be taught to the class by lecture, once every week, to the end. Now, why should I not be glad, if Dr. Lummis will permit me?

To be sure, there is a slight chill comes over our hilarity in the matter of this last college, from a cause which I think no reasonable mind ever could have foreseen or imagined. Almost at the same time the class were assured that the *Organon* would be taught once a week (which, by the way, it had never been before), the faculty joined the class in announcing, as an advance in the right direction on its past history, and an improvement to boast of, that to one of its professors had been given the duty of teaching weekly (just the time they were to devote to the *Organon*) the preparation of *pukes*, *purges*, and *poultices*, three nuisances from which we have these many years regarded the *Organon* as having emancipated the sick-room. This could not be otherwise than a damper on our expectations of good from the promised teaching of the *Organon*, as this was to come from a faculty, so utterly oblivious of the alien nature of these appliances of an antiquated age and school to all which is characteristic of, or pertaining to, the specific system of medicine contained in the *Organon*. Surely a faculty must be wholly unfitted, in its mental make up, for the promised instruction of the philosophy of the therapeutics of the *Organon*, before an idea so absurd as that these nuisances could, by possibility, add aught of value to a knowledge of this philosophy. The suggestion of this brings conviction of utter absence of all ideas as to the nature of the letter or spirit of this philosophy, which they have promised to teach. How can they teach that of which they here show so perfect ignorance of its nature and scope? Evidently they cannot do this, unless the ingenuity which has intruded these wholly unneeded nuisances into the curriculum of a *homœopathic* college, can invent a method by which a man (or a faculty) can teach that which he does not himself possess.

Did the suggestion of this addition to their curriculum spring from an internal consciousness of defective knowledge of homœopathic therapeutics, and that this necessitated the presentation of a something to fall back on when this defect should appear in clinical failures? If so, we cannot praise the felicity of the choice of the means proposed as an escape from this embarrassment. We have said these resorts are not needed in a homœopathic therapeutics. In a practice of this system now covering a period of more than forty years, we have seen not *one* occasion for their use, or in any one instance felt any need of aught they could give of any aid to the sick. And this has not been because of any want of experience of these. We had more than enough of this when we knew no better.

Was it this conscious need of something to fall back on that gave the duty of teaching pukes, purges, and poultices, to a professor, or was this thrown into the curriculum as a counterpoise of the promise to teach the *Organon* once a week? Was it from a desire to assure timid minds that no so very great departure from old physic was intended as should in any way interfere with this faculty joining it "*on a scientific basis*"? They, seemingly, would not have this scanty recognition of the claims of the *Organon* in any way interfere to discourage any hopes which might be looking for a near junction on this basis.

Did this faculty ask themselves why their college was created, and they placed in it as teachers? Was there any other reason for either than that students might there be taught the philosophy and therapeutics of the *Organon?* If there has been any other reason we have not known it. Were they so conscious of inability to discharge this duty that they felt constrained to supplement their labors in this, their specialty, by adding thereto a knowledge of pukes, purges, and poultices? If so, we are impelled to remind them that the supplement is in no way equal or adapted to supplying the deficiency. If this is the outcome of *improvement* in "medical education" or in our college curriculum, we cannot resist the conviction that it would be better if our colleges should cease to be. In view of this discouragement, we are sorry to say we cannot be so glad as we would like to be before the announcement that the *Organon* would be taught once a week. We cannot be, for the reason we cannot see how minds capable of so great absurdity as is this proposed addition to a *homœopathic* curriculum can teach the philosophy of Homœopathy at all.

P. P. WELLS.

